# Some Mysterious Cases

**Published:** 1899-12

**Authors:** D. W. Barker

**Affiliations:** Brooklyn, N. Y.


ARTICLE II.

### SOME MYSTERIOUS CASES.

D. W. BARKER, M. D. S., BROOKLYN, N. Y.

The cases described below have occurred during the past few years, and though at the time I thought I could guess at the cause of some of them, the absence of confirmative data in the others has led me to doubt the correctness of my guess.

A lady about forty-five years of age, with the most perfect denture I think I ever saw, consulted me in regard to an abscess, the fistula of which was in the site of the left upper second bicuspid, that tooth having been lost. Exploration failed to find any remains of the bicuspid root and the adjoining teeth were normal. Endeavoring to find the cause I at last concluded that the pulp of the first bicuspid was dead. This diagnosis was made more because of the absence of any other data than anything connected with that particular tooth, though I may add that it was slightly tender. Acting on this diagnosis I started to drill through the crown; the drill had hardly penetrated the enamel when the patient gave evidence that it was unmistakable alive. Of course I drilled no further and no harm had been done by my error. I treated the fistula with the usual remedies for some time, and making no progress I consulted her physician. Having a supersensitive professional science he almost refused to talk about the case, and the utmost satisfaction I could get was the assurance that I "never would cure that abscess." He was right; I did not. The patient subsequently died under circumstances that led me to believe that she had had syphilis, but whether the abscess was caused by the syphilis or the mercurial treatment I never knew.

A widow about sixty years of age presented with considerable swelling and pain over the right superior central and lateral incisors; both teeth were very loose and sore. I diagnosed a dead pulp in the central as that had a filling in it and the lateral was intact. I lanced the gum and pus flowed freely and the swelling subsided in a few days, though the teeth continued loose. A week later I drilled into the central, the teeth being so loose that I incased them in cement to hold them firm. To my surprise I found the pulp alive. I then tested the rest of the teeth with ice and they all showed live pulps. I was puzzled; there was the abscess plain enough, there was no mistake about that, but where was the cause ? In the course of a week a similar though smaller abscess formed on the palatal gum near the first and second molars. A series of questions brought out the following facts : A few weeks before I saw her she had had a similar "sore spot," as she called it, in the roof of the mouth; the teeth had suddenly become loose; there was a profuse flow of saliva and a strong metallic taste in the mouth. Her ordinary health was good. Seventeen years previous she had been dosed with calomel for the purpose of relieving biliary calculus. Recently she had become "run down" in health to the extent that her physician had put her on a treatment of tonics. These facts together with remarks she reported made by the physician at the time caused me to conclude that the mercury administered seventeen years before had lain dormant in her system until a favorable condition for their development arrived and then this train of symptoms suddenly sprung into activity. Could this be true ? In this case I had no reason to suspect the presence of syphilis, and if it was not the mercury what was it ? Although the teeth became a little firmer when the inflammation subsided they never regained their former state of health and I subsequently extracted them when they were so loose as to be easily removed with my fingers; there has been no recurrence of the abscesses so far as I know in the last two years.

A lady of about forty-five years of age complained of severe pain and swelling in the region of the left lower lateral and cuspid; neither tooth had either cavity or filling. An abscess was apparently developing and as the lateral was loose and sore I diagnosed a dead pulp. I started to drill into it and found the pulp alive and ceased drilling. The abscess continued to form in spite of treatment and a day or two later it was lanced. On the subsidence of the acute inflammation the pulp was found to be still alive; my drill, however, had gone too close and it was deemed best to devitalize it, which was done, and the tap hole filled. In this case no history of syphilis or mercurial treatment could be found, though I think such a course of treatment might have been followed and the patient not have been aware of it. The symptoms were exactly those of the other cases and I cannot help thinking had a similar origin. A strange fact is that the abscess disappeared almost as quickly as it came without any treatment, the teeth regained their natural degree of comfort and are still retained.

What was the cause of these mysterious abscesses? *Quien sabe?—Items of Interest.*

